# Occurrence and Characterization of *Verticillium alfalfae* Causing Alfalfa Verticillium Wilt in Inner Mongolia, China, with Preliminary Fungicide Sensitivity Assessment

**DOI:** 10.3390/microorganisms14071394

**Published:** 2026-06-24

**Authors:** Luran Wang, Ruifang Jia, Na Wang, Shengze Wang, Yuanyuan Zhang, Kejian Lin, Jun Zhao

**Affiliations:** 1 Key Laboratory of Biohazard Monitoring and Green Prevention and Control for Artificial Grassland, Ministry of Agriculture and Rural Affairs and Inner Mongolia Key Laboratory of Grassland Conservation Ecology, Institute of Grassland Research of CAAS, Hohhot 010010, China; 15848189968@163.com (L.W.); jiaruifang@caas.cn (R.J.); 15764743991@163.com (N.W.); wangzhezhe96@163.com (S.W.); linkejian@caas.cn (K.L.); 2 College of Horticulture and Plant Protection, Inner Mongolia Agricultural University, Hohhot 010018, China; zhaojun@imau.edu.cn

**Keywords:** *Verticillium alfalfae*, alfalfa, Inner Mongolia, fungicide sensitivity, virulence, disease survey

## Abstract

Alfalfa Verticillium wilt, caused by *Verticillium alfalfae*, is a globally significant disease with increasing incidence and expanding epidemic areas. This study surveyed six major alfalfa-producing regions in Inner Mongolia, China—Chifeng, Tongliao, Ulanqab, Ordos, Bayannur, and Hohhot—and successfully isolated *V. alfalfae* exclusively from samples collected in Hohhot and Bayannur. Based on morphological characterization, multi-locus phylogenetic analysis (*act*, *tef1-α*, *gapdh*, and *ts* genes), and pathogenicity tests fulfilling Koch’s postulates, all 33 isolates were consistently identified as *V. alfalfae*, with disease severity levels ranging from 3.04 to 4.79 on the susceptible cultivar Zhongmu No. 1. As a preliminary assessment, the in vitro sensitivity of a representative strain, Va8, to eight commercial fungicides was evaluated using the mycelial growth inhibition method. Among the tested fungicides, 30% difenoconazole–propiconazole exhibited the strongest inhibitory effect (EC_50_ = 0.14 μg/mL), followed by 10% trifloxystrobin & 20% tebuconazole (EC_50_ = 0.20 μg/mL). However, given the substantial virulence variation observed among isolates, these sensitivity data should be interpreted with caution, as population-level differences may exist. These findings represent the first confirmed report of *V. alfalfae* in Inner Mongolia and provide a preliminary yet critical reference for prioritizing candidate fungicides for future multi-isolate and field evaluations.

## 1. Introduction

Alfalfa (*Medicago sativa* L.) is a perennial herbaceous plant of the Leguminosae family, native to Asia Minor, Transcaucasia, and Iran. Renowned for its high protein, vitamin, mineral, and essential amino acid contents, alfalfa is globally recognized as the “King of Forages” and serves as a vital nutritional source for livestock and humans [1]. China has emerged as the world’s second-largest alfalfa producer, with large-scale commercial forage production clusters in Gansu, Inner Mongolia and Ningxia [2,3]. Among these, Inner Mongolia has become an increasingly strategic production base. The health and productivity of these alfalfa production areas are of considerable economic significance, and any emerging threat to crop health warrants vigilant investigation.

Alfalfa Verticillium wilt is a devastating vascular disease first reported in Sweden in 1918 [4] and subsequently documented across Europe and North America [5,6], underscoring the pathogen’s capacity for long-distance dissemination and its adaptability to diverse agroecological zones. In China, the disease was first identified in 1996 in Xinjiang [7], with further outbreaks reported across multiple prefectures by the early 2000s [8]. By 2014, the disease had reached Gansu Province, where it spread rapidly within local alfalfa production systems [9], and by 2024 it had become prevalent throughout the Hexi Corridor, a major commercial alfalfa production area in China [10]. Despite this clear eastward range expansion, no confirmed occurrences had been reported in Inner Mongolia prior to the present study. Given the region’s proximity to affected Gansu production areas and the frequent movement of forage products and seeds across provincial boundaries, the absence of surveillance data from Inner Mongolia represented a critical gap in understanding this pathogen’s distribution.

The causal agent was originally described as *Verticillium albo-atrum* in 1879 based on morphological features [11], but molecular phylogenetic studies by Inderbitzin et al. [12] reclassified the alfalfa pathogen as the distinct species *V. alfalfae* (*Ascomycota*, *Sordariomycetes*, *Hypocreales*). *V. alfalfae* predominantly infects leguminous plants, causing characteristic symptoms including “V”-shaped chlorosis, progressive foliar chlorosis and necrosis, vascular discoloration, and ultimately plant wilting and death, with infected stems often remaining green and upright even after foliar collapse [13]. The pathogen also infects various vegetable crops and common weeds [14], complicating disease management through potential inoculum reservoirs in non-crop vegetation.

The damage inflicted by *V. alfalfae* has become a serious issue in China, where alfalfa production has intensified to meet growing demands from the dairy and livestock sectors. Once established, the pathogen can persist in soil through its resting mycelium and spreads via irrigation water, rain splash, insect vectors, contaminated equipment, and infected plant material [5]. Management options are limited by the scarcity of resistant cultivars, impractical rotation requirements, and the difficulty of eradicating soilborne inocula. Chemical fungicides thus represent a key component of integrated disease management, though their rational application requires baseline sensitivity data to avoid resistance development and environmental risks [15]. Although research on Alfalfa Verticillium wilt has been conducted in Xinjiang and Gansu [16], comprehensive pathogen identification and characterization data for Inner Mongolia remain entirely absent. This gap is particularly concerning given that Inner Mongolia has emerged as one of China’s most important alfalfa-producing regions. Without confirmed pathogen identification and baseline fungicide sensitivity data, local producers lack evidence-based guidance for effective disease management. Furthermore, the potential spread of the pathogen from adjacent affected areas in Gansu represents a tangible biosecurity threat requiring proactive investigation.

To address these knowledge gaps, the present study was designed to: (1) conduct a systematic disease survey across major alfalfa-producing regions in Inner Mongolia and identify the causal agent using morphological characterization, multi-locus phylogenetic analysis (*act*, *tef1-α*, *gapdh*, and *ts* genes), and pathogenicity assays and (2) evaluate the in vitro sensitivity of the identified pathogen to eight commercial fungicides, establishing preliminary baseline efficacy data for local disease management. These results provide the first confirmed report of *V. alfalfae* in Inner Mongolia and a foundation for developing region-specific disease management strategies.

## 2. Materials and Methods

### 2.1. Sample Collection and Pathogen Isolation

A disease survey was conducted across six major alfalfa-producing regions in Inner Mongolia—Chifeng, Tongliao, Ulanqab, Ordos, Bayannur, and Hohhot—from June to August 2024. A total of 54 alfalfa fields were selected using quota-based random sampling proportional to the cultivation area of each region. In each field, a diagonal five-point sampling method was employed along a Z-shaped transect. At each of the five equidistant points, all plants within a 1 m^2^ quadrat or 30–50 randomly selected plants were examined, with a minimum of 150 plants assessed per field. Plants exhibiting typical symptoms of *Verticillium alfalfae* infection, including progressive yellowing and wilting from lower to upper leaves, V-shaped necrotic lesions at leaf tips, and twisting of young leaves, were scored as diseased. Disease incidence was expressed as the percentage of symptomatic plants among the total surveyed plants. Symptomatic stem base and crown tissues were collected from all six regions, transported at low temperature, and subjected to pathogen isolation and identification. Based on this preliminary field estimation, disease incidence ranged from 0% to 10% across the surveyed fields. However, *Verticillium alfalfae* was successfully isolated only from the symptomatic samples collected in Hohhot City (Tumd Left Banner) and Bayannur League (Dengkou County). Details of the 33 isolates recovered from these two regions are listed in Table 1.

Diseased tissues from the junction of symptomatic and healthy areas of leaves, petioles, stems, and roots were cut into 3 mm × 3 mm pieces. These pieces were surface-sterilized in 70% ethanol for 5 s, followed by 0.5% sodium hypochlorite for 1 min, rinsed three times in sterile distilled water, and dried on sterile filter paper. Tissue slices were then plated on 1.5% water agar (WA: 15 g agar per liter of distilled water) and incubated at 25 °C for 7 days. Hyphal tips were transferred to a potato dextrose agar plate (PDA: 40 g potato dextrose agar powder per liter of distilled water) to obtain pure cultures. After 10 days of incubation at 25 °C, colony morphology, conidiophores, and conidia were observed and photographed using the Nikon H550s camera system (Nikon, Tokyo, Japan). Isolates were then stored at −80 °C in a 50% glycerol–water solution.

### 2.2. DNA Extraction, Amplification, Sequencing, and Phylogenetic Analyses

Genomic DNA was extracted from fresh mycelia using a rapid plant DNA extraction protocol [17]. Briefly, fresh mycelia were scraped from the surface of 7-day-old PDA cultures using a sterile pipette tip and transferred to a PCR tube containing 50 μL of Buffer A (2% Tween-20, 100 mM NaOH, freshly diluted from stock solutions). The tube was incubated at 95 °C for 10 min in a thermal cycler to facilitate cell lysis. Subsequently, 50 μL of Buffer B (100 mM Tris-HCl, 2 mM EDTA-Na_2_, pH 8.0) was added and mixed thoroughly by gentle vortexing. The crude DNA extract was centrifuged at 12,000 rpm for 1 min, and the resulting supernatant containing genomic DNA was transferred to a new sterile tube and stored at −20 °C for subsequent use.

PCR amplifications targeting four nuclear loci—Actin (ACT), Glyceraldehyde-3-phosphate dehydrogenase (GPD), Tryptophan synthase (TS), and elongation factor 1-α (EF)—were conducted in 25 μL reaction volumes. Each reaction contained 12.5 μL of 2× Taq PCR Mix (TIANGEN Biotech, Beijing, China), 1 μL of genomic DNA, 1 μL each of the forward and reverse primers (10 μM), and 9.5 μL of sterile double-distilled H_2_O. The primer pairs and their respective annealing temperatures are detailed in Table 2. The PCR thermal cycling program consisted of an initial denaturation step at 94 °C for 5 min, followed by 35 cycles of denaturation at 94 °C for 40 s, annealing at 48–60 °C (optimized for each gene as specified in Table 2) for 40 s, and extension at 72 °C for 40 s, with a final extension step at 72 °C for 10 min. The specificity and yield of the amplified products were verified by electrophoresis on 1.5% (*w*/*v*) agarose gels in 1 × TAE buffer, stained with ethidium bromide, and visualized under UV illumination using a QI G5000 gel imaging system (Daoyi, Guangzhou, China). The amplicons were sequenced by Tsingke Biotechnology Co., Ltd., to ascertain the fungal species present. All newly generated sequences of the *act*, *tef1-α*, *gapdh*, and *ts* gene regions have been deposited into the NCBI GenBank database. For phylogenetic analyses, the newly generated sequences were compared against the NCBI GenBank nucleotide database using the BLASTn (NCBI, https://blast.ncbi.nlm.nih.gov/Blast.cgi, accessed on 15 December 2024) algorithm to retrieve closely related reference sequences from taxonomically verified *Verticillium* species. Sequences of representative *Verticillium* spp., including *V. dahliae*, *V. albo-atrum*, *V. tricorpus*, *V. longisporum*, *V. klebahnii*, *V. nubilum*, *V. zaregamsianum*, and *V. nonalfalfae*, as well as the outgroup taxon *Gibellulopsis nigrescens*, were downloaded from GenBank. Individual sequence alignments for each of the four gene regions were performed using the MAFFT v.7 online server employing the L-INS-i iterative refinement algorithm [18]. The resulting alignments were manually inspected and trimmed at both ends to remove ambiguous regions using MEGA 11.0 [19]. The four single-gene alignments were subsequently concatenated into a combined multi-locus dataset using SequenceMatrix v.1.8 [20]. A Maximum Likelihood (ML) phylogenetic tree was constructed using MEGA 11.0 with 1000 bootstrap pseudo-replicates to assess branch support.

### 2.3. Pathogenicity Assay

Pathogenicity tests were conducted for all 33 isolates, along with the reference strain *V. alfalfae* Ms198 (kindly provided by Professor Yanzhong Li, Lanzhou University), on the susceptible alfalfa cultivar Zhongmu No. 1. Conidial suspensions were prepared from pure cultures grown on PDA plates at 25 °C for 14 days. To harvest conidia, sterile distilled water (approximately 10 mL per plate) was added to the culture surface, and the mycelial mat was gently scraped with a sterile glass spreader to dislodge the conidia. The resulting suspension was filtered through four layers of sterile gauze to remove mycelial fragments. The conidial concentration was determined using a hemocytometer and adjusted to a final concentration of 1 × 10^7^ conidia/mL with sterile distilled water.

Healthy alfalfa seeds (cv. Zhongmu No. 1) were surface-sterilized by immersion in 70% ethanol for 30 s, followed by 1% sodium hypochlorite for 3 min, and then rinsed thoroughly five times with sterile distilled water. The sterilized seeds were germinated on moistened sterile filter paper in Petri dishes at 25 °C in darkness for four days. Uniformly germinated seedlings at the cotyledon stage were selected and transplanted into plastic pots (diameter × height = 13 cm × 13 cm) filled with 100 g of sterilized soil (autoclaved twice at 121 °C for 60 min on consecutive days), with six seedlings per pot. The transplanted seedlings were maintained in a growth chamber at 25 ± 2 °C with a 16 h photoperiod (approximately 300 μmol photons m^−2^ s^−1^) and 60–70% relative humidity and were watered as needed with sterile distilled water for two weeks to allow establishment before inoculation. At two weeks post-transplantation, inoculation was performed using the root-drenching method. Each pot was inoculated by evenly pouring 100 mL of the conidial suspension (1 × 10^7^ conidia/mL) onto the soil surface around the bases of the seedlings. Control plants were treated with an equal volume (100 mL) of sterile distilled water. Each isolate was tested in three replicate pots (18 seedlings per isolate in total), and the experiment was conducted twice under identical conditions to confirm reproducibility.

Following inoculation, plants were maintained under the same controlled environmental conditions as those described above. The pots were arranged in a completely randomized design within the growth chamber and re-randomized weekly to minimize positional effects. Plants were monitored weekly for symptom development. At 30 days post-inoculation (dpi), disease severity was assessed for every individual plant using a standardized 1–5 rating scale adapted from Christen et al. [6], where: 1 = no visible symptoms; 2 = one or two leaves showing chlorosis; 3 = numerous leaves exhibiting chlorosis with the plant showing evidence of stunting or significant damage; 4 = more than 50% of the total foliage displaying chlorosis or necrosis; and 5 = dead plant. Based on the disease severity rating, the disease severity level (DSL) for each treatment was calculated using the formula:

DSL = ∑(n_i_ × s_i_)/N

where n_i_ represents the number of plants assigned to each severity grade, s_i_ represents the numerical value of that severity class (1 to 5), and N represents the total number of plants assessed (including both symptomatic and healthy plants) in the treatment group.

To confirm Koch’s postulates, symptomatic tissues were collected from inoculated plants at 30 dpi and subjected to re-isolation using the same protocol described in the pathogen isolation section. Morphological and molecular identification of the re-isolated fungi was performed to verify that the recovered pathogen was identical to the original inoculated strain.

To compare the virulence of the 33 isolates, the mean DSL value for each isolate was calculated from the three replicate pots. The pathogenicity assay was performed twice independently under identical conditions to confirm reproducibility. The results of both runs were highly consistent, with no substantial differences in disease severity levels or isolate ranking observed between the two experiments. Therefore, the data from one representative experimental run are presented and analyzed herein. Differences in virulence among isolates were analyzed using one-way analysis of variance (ANOVA), and mean separations were performed using Duncan’s multiple range test at significance levels of *p* < 0.05 and *p* < 0.01.

### 2.4. In Vitro Fungicide Sensitivity Assay

As a preliminary assessment of chemical control options, the in vitro sensitivity of *V. alfalfae* to eight commercial fungicides was evaluated using the mycelial growth inhibition method. Strain Va8, which exhibited moderate–high virulence among the 33 isolates (DSL = 4.625), was selected as a representative strain for this initial screening. Despite the likelihood of differential sensitivity across the population given the virulence variation observed, establishing baseline efficacy against a single representative strain remains a necessary and valuable first step. The eight fungicides tested, along with their formulation types, registration numbers, and the concentration ranges used in the assay, are listed in Table 3. Fungicide concentration gradients were determined based on the field application rates specified in each product’s manufacturer instructions, with preliminary range-finding experiments conducted to ensure the selected concentrations produced inhibition rates spanning from approximately 10% to 90%, thereby enabling reliable EC_50_ estimation.

Stock solutions of each fungicide were freshly prepared in sterile distilled water at concentrations 100-fold higher than the highest working concentration for water-dispersible formulations. PDA medium was autoclaved and cooled to approximately 50 °C in a water bath before the addition of the fungicide stock solutions. The medium and fungicide solution were thoroughly mixed by gentle swirling to ensure uniform distribution of the fungicide throughout the medium, and approximately 20 mL of the amended medium was poured into each sterile 90 mm diameter Petri dish. Plates were allowed to solidify at room temperature for at least 2 h in a laminar flow hood before inoculation. Each fungicide concentration was tested in five replicates (five plates per concentration). Unamended PDA plates, prepared with an equivalent volume of sterile distilled water, served as the negative control. All fungicide formulations (SC, EC, WP, WG, and SD) were diluted directly in sterile distilled water without the addition of organic solvents.

To prepare the inoculum, strain Va8 was cultured on PDA plates at 25 °C for 7 days in darkness. Mycelial plugs (8 mm in diameter) were aseptically excised from the actively growing margin of the colony using a sterile cork borer. A single mycelial plug was transferred, mycelial side down, to the center of each fungicide-amended or control PDA plate. All inoculated plates were sealed with Parafilm to minimize evaporation and incubated in the dark at 25 °C. The colony diameter on each plate was measured daily using a digital caliper in two perpendicular directions (cross-intersection method), and measurements were continued until the colony in the control treatment reached approximately two-thirds to three-quarters of the plate diameter (typically 7–8 days post-inoculation), ensuring that growth remained in the linear phase and was not constrained by plate edge effects.

The percentage of mycelial growth inhibition (I) for each fungicide concentration was calculated using the formula:

I (%) = [(D_c_ − D_t_)/(D_c_ − D_0_)] × 100

where D_c_ represents the mean colony diameter (mm) of the control treatment, D_t_ represents the mean colony diameter (mm) of the fungicide-treated plates, and D_0_ represents the initial diameter of the mycelial plug (8 mm). For each fungicide, the logarithm of the fungicide concentration (x-axis) was plotted against the probit-transformed values of the percentage inhibition (y-axis). The half-maximal effective concentration (EC_50_), defined as the fungicide concentration required to inhibit mycelial growth by 50% relative to the control, was calculated from the linear regression equation (y = a + bx) by substituting y = 5.000 (the probit value corresponding to 50% inhibition). The coefficient of determination (R^2^) was used to evaluate the goodness-of-fit of the regression model. The entire experiment was repeated twice independently to confirm reproducibility, and the reported EC_50_ values represent the mean of both independent experiments.

## 3. Results

### 3.1. Symptoms, Incidence, and Distribution of Verticillium Wilt

**Figure 1:**
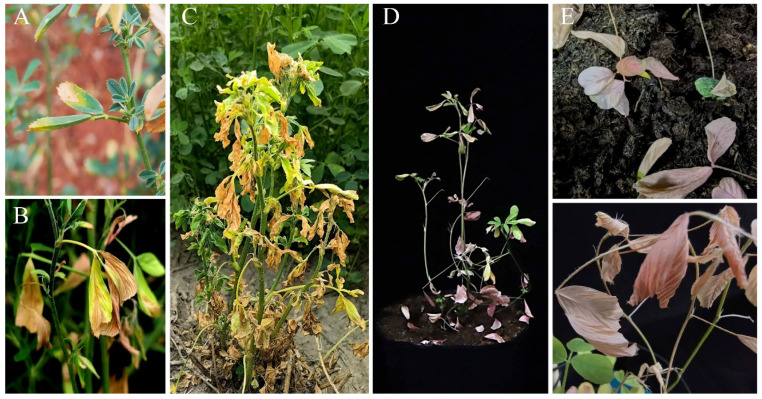
Symptoms of Verticillium wilt of alfalfa. (**A**) Early-stage leaves showing chlorosis and yellow spots. (**B**) Expanded chlorotic lesions from leaf margins and midrib, forming characteristic V-shaped patterns. (**C**) Severely diseased plant with wilted and necrotic foliage; note the green, upright stem. (**D**,**E**) Curled, detached senescent leaves with yellow–brown to orange–pink discoloration.

During the 2024 growing season, a systematic disease survey was conducted across six major alfalfa-producing regions in Inner Mongolia: Chifeng, Tongliao, Ulanqab, Ordos, Bayannur, and Hohhot. Alfalfa plants exhibiting symptoms characteristic of Verticillium wilt were observed in all six regions. However, *V. alfalfae* was successfully isolated only from symptomatic samples collected in Hohhot City (Tumd Left Banner) and Bayannur League (Dengkou County), where the disease incidence ranged from 1% to 10% at the field level. Although symptomatic plants were also observed in Chifeng, Tongliao, Ulanqab, and Ordos, repeated isolation attempts from these regions failed to recover *V. alfalfae*. No other consistent fungal pathogen was identified from these samples, indicating that the observed symptoms could not be definitively attributed to any single biological agent and may have resulted from other biotic or abiotic factors. Initial symptoms appeared as small yellow spots on the leaves, which gradually expanded along the midrib and veins, forming yellow stripes. These patterns developed on the leaves of individual stems and occasionally on the leaves of several stems of the same plant. As the infection progressed, a characteristic “V”-shaped chlorosis developed at the tips of the leaflets (Figure 1A,B). Severely infected plants showed pronounced wilting and eventually died, although certain stems often remained green, upright, and apparently healthy even after the foliage had collapsed (Figure 1C). All infected leaves ultimately underwent necrosis and desiccation. Some necrotic leaves remained attached to the green stems by their petioles, while others abscised and fell to the soil surface, exhibiting a conspicuous yellow–brown to orange–pink discoloration (Figure 1D,E).

### 3.2. Pathogen Isolation and Morphological Characterization

**Figure 2:**
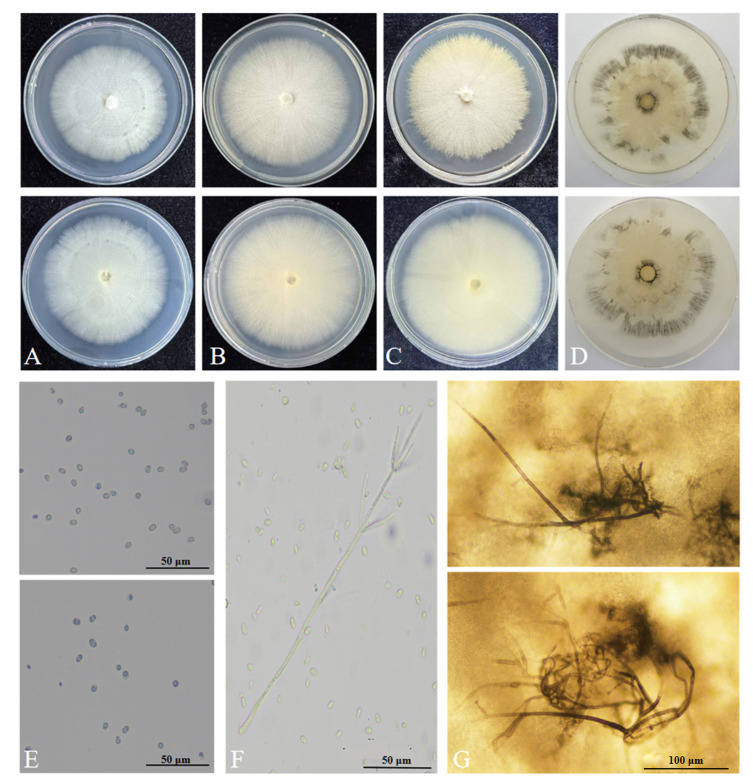
Morphological characteristics of *Verticillium alfalfae*. (**A**) Colony morphology on PDA at 5 days post-inoculation, showing a white, smooth appearance. (**B**,**C**) Colonies at 10–14 days, displaying pale yellow coloration with alternating light and dark concentric rings. (**D**) Aging colonies (21 days) with dark-gray pigmentation due to dormant mycelium formation. (**E**) Hyaline, aseptate, cylindrical to ellipsoidal conidia. (**F**) Verticilliate conidiophores with whorls of phialides. (**G**) Brown to dark brown dormant hyphae forming compact masses. Scale bars in (**E**,**F**) = 50 μm, (**G**) = 100 μm.

A total of 33 pure cultures were obtained via monoconidial isolation from the basal stems of diseased alfalfa samples collected from the six sampling locations in Hohhot and Bayannur (Table 1; Supplementary Figure S1). All 33 isolates obtained exhibited consistent morphological features when cultured on PDA at 25 °C. Colonies were initially white, smooth and flat with even margins (Figure 2A,B). After approximately 5–7 days of incubation, the colonies gradually developed a pale yellowish tint (Figure 2C–F). The colony surface was characterized by the formation of alternating light and dark concentric rings, and the texture remained homogeneous. With prolonged incubation (14–21 days), the colonies progressively darkened in color due to the formation of dormant mycelium, eventually turning pale gray to dark gray (Figure 2G,H). Conidiophores were hyaline, erect, and septate, with some exhibiting a slightly darkened base. They were branched in whorls, with each whorl containing 1–5 phialides. Conidia were produced apically from the phialides and were hyaline, aseptate, and cylindrical to ellipsoidal in shape, measuring 7.5–15 μm × 4.2–6.3 μm (Figure 2I,J). The dormant hyphae appeared brown to dark brown and readily aggregated into compact, irregular masses (Figure 2L,M). No microsclerotia were found in any of the isolates, a feature consistent with the description of *V. alfalfae* and distinguishing it from *V. dahliae*.

### 3.3. Sequence and Phylogenetic Analyses

**Figure 3:**
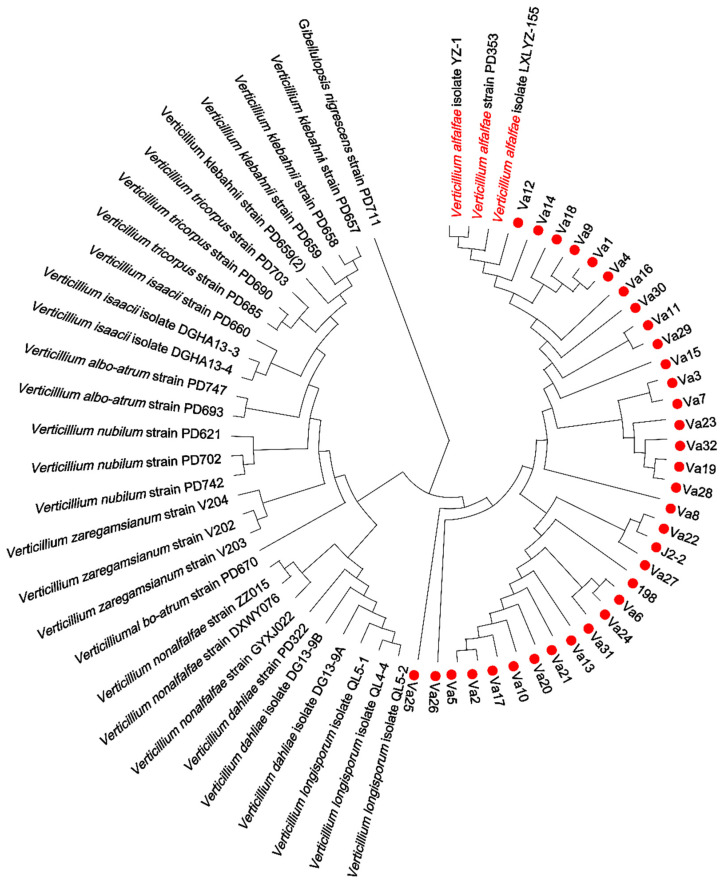
Maximum Likelihood phylogenetic tree of *Verticillium* spp. based on concatenated *act*, *tef1-α*, *gapdh*, and *ts* gene sequences, constructed in MEGA 11.0 with 1000 bootstrap replicates. The 33 isolates obtained in this study (highlighted) cluster within the *V. alfalfae* clade with 99% bootstrap support. *Gibellulopsis nigrescens* was used as the outgroup taxon. Bootstrap support values ≥ 70% are indicated at the nodes. The scale bar represents the number of nucleotide substitutions per site.

The nucleotide sequences of the *act*, *tef1-α*, *gapdh*, and *ts* gene regions were successfully amplified and sequenced for all 33 isolates. The obtained sequences were compared with reference sequences of representative *Verticillium* species retrieved from the NCBI GenBank database. A multi-locus phylogenetic tree was constructed based on the concatenated alignment of the four genes using the Maximum Likelihood method. In the resulting phylogeny, all 33 isolates from Inner Mongolia formed a strongly supported monophyletic clade together with the reference sequences of *V. alfalfae*, with a bootstrap support value of 99% (Figure 3). This clade was clearly distinct from those of other closely related *Verticillium* species, including *V. dahliae*, *V. albo-atrum*, *V. longisporum, V. klebahnii, V. tricorpus, V. nubilum, V. zaregamsianum* and *V. nonalfalfae*. The molecular identification, combined with the morphological characterization described above, unequivocally confirmed that all 33 isolates obtained in this study belong to the species *V. alfalfae*.

### 3.4. Pathogenicity Assay Results

**Figure 4:**
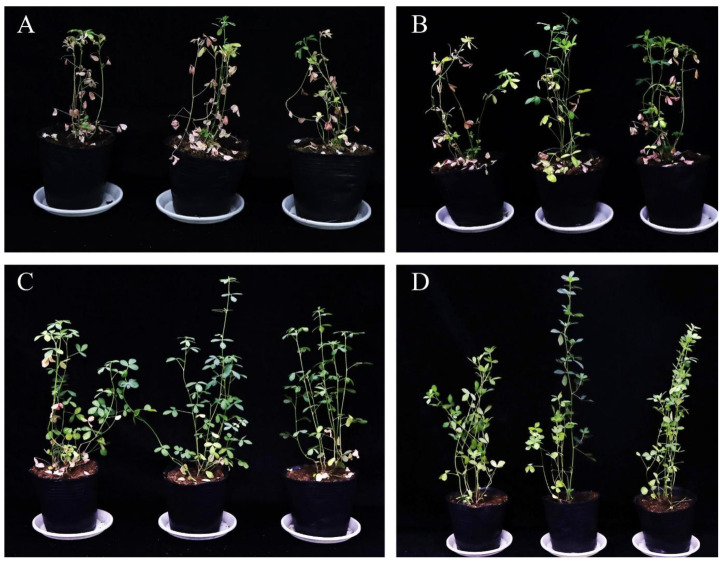
(**A**) Plant inoculated with the highly virulent strain Va23, showing extensive chlorosis, necrosis, and wilting. (**B**) Plant inoculated with the moderately virulent strain Va8, exhibiting characteristic V-shaped chlorosis and partial wilting. (**C**) Plant inoculated with the weakly virulent strain Va30, displaying mild foliar chlorosis. (**D**) Control plant treated with sterile water, showing healthy growth with no visible symptoms.

The pathogenicity of the 33 isolates, together with the reference strain *V. alfalfae* Ms198, was evaluated on the susceptible alfalfa cultivar Zhongmu No. 1 using a root-drenching inoculation method. All 33 isolates and the reference strain were able to induce disease symptoms on the inoculated seedlings, thereby fulfilling Koch’s postulates. In contrast, the negative control plants treated with sterile distilled water remained completely asymptomatic throughout the entire 30-day trial period. The first visible symptoms, consisting of leaf yellowing and mild wilting, appeared approximately 14 days post-inoculation (dpi), and the disease severity level was recorded at 30 days post-inoculation (dpi). However, significant differences in virulence were observed among the isolates (Table 4, Figure 4). Strain Va23 exhibited the strongest pathogenicity, with a disease severity level (DSL) of 4.792, causing extensive chlorosis and plant death in the majority of inoculated seedlings. In contrast, strain Va30 showed the weakest pathogenicity, with a DSL of 3.042, producing only moderate foliar chlorosis and limited wilting. The reference strain *V. alfalfae* Ms198 had a DSL of 4.333. The majority of the Inner Mongolia isolates (27 out of 33) were classified as moderately to strongly pathogenic, with DSL values ranging from 3.9 to 4.6. The complete results of the statistical comparison of virulence among all isolates, including significant differences at *p* < 0.05 and *p* < 0.01 as determined by Duncan’s multiple range test, are presented in Table 4. Representative disease symptoms induced by the strongly pathogenic strain Va23, the moderately pathogenic strain Va8, the weakly pathogenic strain Va30, and the water-treated control are illustrated in Figure 4.

Data are presented as mean ± standard deviation (*n* = 6). Means followed by the same letter within a column are not significantly different at the indicated probability level according to Duncan’s multiple range test. DSL was calculated as DSL = ∑(n_i_ × s_i_)/N, where n_i_ is the number of plants in each severity class, s_i_ is the severity value (1–5 scale), and *N* is the total number of plants assessed.

### 3.5. In Vitro Sensitivity of Verticillium alfalfae Strain Va8 to Eight Fungicides

The in vitro sensitivity of the representative strain Va8 to eight commercial fungicides was assessed using the mycelial growth inhibition method. All eight fungicides inhibited mycelial growth of *V. alfalfae* in a concentration-dependent manner. However, substantial differences in efficacy were observed among the tested products, with EC_50_ values ranging from 0.14 μg/mL to 288.40 μg/mL (Table 5). Among the eight tested fungicides, 30% Difenoconazole–Propiconazole exhibited the highest inhibitory activity, with an EC_50_ value of 0.14 μg/mL (R^2^ = 0.9253) and the steepest slope in the concentration–response regression (1.9952). This was followed by 10% Trifloxystrobin & 20% Tebuconazole (EC_50_ = 0.20 μg/mL, R^2^ = 0.9975) and 32.5% Fluoxapiprolin & Difenoconazole (EC_50_ = 0.47 μg/mL, R^2^ = 0.9896), both of which also demonstrated strong inhibitory effects. Three fungicides displayed moderate activity: 32.5% Difenoconazole (EC_50_ = 0.62 μg/mL), 15% Triadimefon (EC_50_ = 9.86 μg/mL), and 70% Mancozeb (EC_50_ = 57.41 μg/mL). The least effective fungicides were 68% Metalaxyl-M & Mancozeb (EC_50_ = 288.40 μg/mL) and 35% Metalaxyl-M & Fludioxonil (EC_50_ = 103.04 μg/mL), which required substantially higher concentrations to achieve 50% inhibition of mycelial growth. The slope of the regression line for 35% Metalaxyl-M & Fludioxonil was the shallowest (0.6332), indicating that *V. alfalfae* exhibited relatively low sensitivity to this fungicide combination across the tested concentration range. The full set of EC_50_ values, regression equations, and correlation coefficients is summarized in Table 5. Representative photographic documentation of the mycelial growth inhibition assay for all eight fungicides at the tested concentrations is provided in Supplementary Figure S2.

## 4. Discussion

The accurate identification of *Verticillium* species has long presented a substantial challenge to plant pathologists due to the considerable overlap in morphological traits, physiological profiles, genetic diversity, and potential mutations, and environmental variables such as temperature, pH, and nutrient availability further complicate species identification [21]. Early attempts to differentiate *V. albo-atrum* from *V. dahliae* relied heavily on the presence of dark resting mycelium versus microsclerotia, a distinction that proved inadequate for resolving the full diversity within the genus [22]. In this study, we confronted this taxonomic complexity directly. Although all 33 isolates from Inner Mongolia exhibited morphological features broadly consistent with *V. albo-atrum* sensu lato—namely hyaline conidiophores with verticillate branching, hyaline cylindrical conidia, and the formation of dark resting mycelium without microsclerotia—definitive species-level identification was achieved only through the integration of multi-locus phylogenetic analysis. The concatenated *act*, *tef1-α*, *gapdh*, and *ts* gene sequences resolved all 33 isolates into a well-supported monophyletic clade with the type strain and reference isolates of *V. alfalfae*, confirming their identity with a confidence that morphology alone could not provide. This finding reinforces the now well-established view that polyphasic approaches combining morphological characterization with multi-gene molecular phylogenetics are essential for robust species delimitation within the genus *Verticillium* [12,23,24].

The confirmation of *V. alfalfae* in Inner Mongolia carries significant phytosanitary and epidemiological implications. Although symptomatic plants were observed across all six surveyed regions—Chifeng, Tongliao, Ulanqab, Ordos, Bayannur, and Hohhot—the pathogen was successfully isolated only from the latter two. This restricted distribution is likely attributable to an interplay of environmental, climatic, sampling-related, and anthropogenic factors. First, environmental and climatic conditions strongly influence pathogen establishment and survival. Hohhot and Bayannur lie within the Hetao Plain and adjacent irrigated agricultural zones, where favorable moisture conditions—supported by the Yellow River irrigation system and relatively higher annual precipitation than the arid western Inner Mongolia plateaus—facilitate pathogen survival and dissemination via surface water movement. Verticillium spp. are known to disseminate efficiently through irrigation networks, and the dense canal systems in Bayannur may have accelerated pathogen introduction and local amplification. By contrast, Chifeng, Tongliao, Ulanqab, and Ordos are situated in cooler, drier semi-arid zones with predominantly rain-fed alfalfa production, shorter growing seasons, and limited water availability, all of which may constrain pathogen establishment and the survival of resting mycelium. Second, sampling-related limitations likely affected recovery rates. The survey involved single-site visits to each field, and in the regions from which *V. alfalfae* was not recovered, disease incidence was generally low (often <2%), reducing the probability of successful isolation from limited sample sets. Additionally, the incubation-based isolation method employed in this study may have lower sensitivity than molecular detection approaches for identifying low-density or latent infections. Third, anthropogenic factors may have shaped the observed distribution. Differences in alfalfa cultivar composition, seed sourcing history, and the extent of commercial seed exchange with affected provinces such as Gansu and Ningxia likely influenced the regional introduction and establishment of the pathogen. Notably, both Hohhot and Bayannur maintain active seed importation links with production areas in Gansu and Ningxia, which may have facilitated pathogen introduction. The isolation of *V. alfalfae* from Bayannur, which lies adjacent to the affected Hexi Corridor in Gansu Province, is consistent with a pattern of gradual eastward expansion along contiguous alfalfa-producing corridors.

Finally, the observation of Verticillium wilt-like symptoms in regions from which *V. alfalfae* was not recovered warrants cautious interpretation. These symptoms may be attributable to a range of alternative causal agents. Other soilborne fungal pathogens—including *Fusarium oxysporum*, *F. solani*, and *Rhizoctonia solani*—are capable of producing foliar chlorosis, necrosis, and wilt symptoms in alfalfa that can be difficult to distinguish from Verticillium wilt based on field symptomatology alone [22,25]. In addition, insect pests, particularly piercing–sucking herbivores such as aphids and leafhoppers, can cause feeding damage that results in localized or systemic chlorosis, stunting, and foliar distortion that may superficially resemble early-stage Verticillium wilt symptoms. This study therefore represents the first confirmed report of *V. alfalfae* in Inner Mongolia and establishes a critical baseline for understanding the pathogen’s current distribution in northern China, against which future range shifts can be measured.

We observed substantial variation in virulence among the 33 isolates on the susceptible cultivar Zhongmu No. 1, with DSL values ranging from 3.04 (Va30) to 4.79 (Va23), a finding with important implications for understanding the pathogenic potential of *V. alfalfae* populations in Inner Mongolia. Although the numerical range of DSL values appears narrow (3.04–4.79), each unit increment on the 1–5 severity scale represents a biologically meaningful transition in disease severity. A DSL of approximately 3.0 corresponds to partial foliar chlorosis with limited growth reduction, whereas values exceeding 4.5 indicate extensive necrosis and plant death, marking a qualitative shift from a manageable infection compatible with plant survival to the total loss of forage productivity. Consequently, even small numerical differences among isolates translate into significant agronomic consequences, directly determining whether infected plants can persist in the field or require immediate removal, crop rotation, or chemical control. We believe the clustering of 27 out of 33 isolates within the moderate-to-high virulence interval (DSL 3.9–4.6) is at least partly attributable to our sampling strategy, which targeted plants exhibiting characteristic Verticillium wilt symptoms in the field. This approach inherently biases the isolate collection toward strains capable of producing visible disease phenotypes under natural field conditions. Highly aggressive strains that cause rapid plant death and complete tissue necrosis may have been overlooked because dead plants were less likely to be sampled during our survey. Conversely, weakly pathogenic strains that induce only mild or asymptomatic infections under field conditions would also have escaped detection, as they did not produce the conspicuous foliar symptoms that guided our sampling. Consequently, the isolates we recovered predominantly represent the moderate-to-strong virulence spectrum of the regional *V. alfalfae* population. From this perspective, the narrow DSL range is itself biologically informative—it suggests that the strains responsible for visible field epidemics in Inner Mongolia share a relatively consistent virulence phenotype. This distribution pattern reveals that the *V. alfalfae* population currently established in Inner Mongolia is predominantly aggressive, which has direct practical implications for resistance breeding: alfalfa germplasm should be screened against representative strains from this prevalent virulence class to ensure the selection of cultivars with durable, regionally adapted resistance. Intraspecific variation in aggressiveness is a well-documented phenomenon in vascular wilt pathogens and has been reported for *V. dahliae* across diverse host systems [26]. Such variation may arise through multiple mechanisms. Genomic comparisons of *Verticillium* species have revealed extensive lineage-specific genomic regions enriched in transposable elements and putative effector genes that are likely to contribute to virulence plasticity [27,28]. Attenuated virulence, as observed in strain Va30, may result from loss-of-function mutations in pathogenicity genes, epigenetic silencing of virulence factors, or genomic rearrangements within effector-rich chromosomal regions during prolonged saprophytic growth or storage. From an ecological perspective, reduced virulence does not necessarily connote reduced fitness: strains with intermediate or low aggressiveness may persist more effectively in the absence of susceptible hosts by allocating resources to saprophytic competence and survival structures, a life-history trade-off that has been demonstrated in other soilborne plant pathogens [26]. Furthermore, the presence of a broad spectrum of virulence within a geographically localized pathogen population suggests that host selection pressure, if applied through the deployment of resistant cultivars, could drive further shifts in population structure over time. These observations underscore the importance of monitoring virulence dynamics as an integral component of resistance breeding and disease management strategies.

The in vitro fungicide sensitivity data obtained in this study provide a preliminary characterization of chemical control options for *V. alfalfae* in Inner Mongolia and reveal clear efficacy differentials among the eight tested products against the representative strain Va8. We emphasize that these data were derived from a single strain and should therefore be interpreted with appropriate caution; multi-isolate testing is required to determine whether the sensitivity profile of Va8 is representative of the broader *V. alfalfae* population in this region. Despite this limitation, the superior performance of 30% Difenoconazole–Propiconazole (EC_50_ = 0.14 μg/mL) is mechanistically plausible and practically significant. Both active ingredients belong to the demethylation inhibitor (DMI) class of fungicides, which target the sterol 14α-demethylase enzyme encoded by the *CYP51* gene family, thereby disrupting ergosterol biosynthesis and compromising fungal cell membrane integrity [29]. Chemical control remains a key component of integrated disease management for fungal plant pathogens [30]. Although the specific CYP51 paralogues of *V. alfalfae* have not yet been experimentally characterized, studies in Fusarium graminearum have demonstrated that multiple CYP51 paralogues (CYP51A, CYP51B, and CYP51C) exhibit functional diversification, with differential roles in sterol demethylation and intrinsic azole sensitivity [31]. Consequently, the enhanced efficacy of the difenoconazole–propiconazole combination observed in this study may reflect differential binding affinities to multiple CYP51 isoforms, should they exist in *V. alfalfae*; however, this hypothesis requires direct genomic and biochemical validation. The steep slope of the concentration–response regression for this product (1.9952) further indicates that small increases in concentration produce disproportionately large gains in efficacy against Va8, a property that has practical advantages in field application. The strong activity of 10% Trifloxystrobin & 20% Tebuconazole (EC_50_ = 0.20 μg/mL against strain Va8) is also noteworthy. This product combines a quinone outside inhibitor (QoI) targeting mitochondrial respiration with a DMI targeting sterol biosynthesis, thereby simultaneously disrupting two essential cellular processes. Multi-site targeting—whether through formulated mixtures or rotational schemes—is a cornerstone of resistance management and is particularly important for vascular wilt pathogens with long latent periods and the potential for repeated exposure to fungicide selection pressure [32].

In contrast, the weak activity of the two Metalaxyl-M-containing products (68% Metalaxyl-M & Mancozeb, EC_50_ = 288.40 μg/mL; 35% Metalaxyl-M & Fludioxonil, EC_50_ = 103.04 μg/mL against strain Va8) is entirely consistent with the known mode of action of this active ingredient. Metalaxyl-M is a systemic fungicide that specifically inhibits RNA polymerase I activity in oomycetes through interference with ribosomal RNA synthesis, and it is consequently ineffective against true fungi belonging to the *Ascomycota*, including *Verticillium* species [33]. The residual moderate inhibition observed with these products is attributable to the mancozeb component, a multi-site contact fungicide that provides broad-spectrum protective activity against a range of fungi, but whose efficacy against deep-seated vascular pathogens is inherently limited by its inability to penetrate xylem tissues at effective concentrations [29]. The similarly moderate performance of 70% Mancozeb alone (EC_50_ = 57.41 μg/mL against strain Va8) corroborates this interpretation.

A critical consideration in interpreting these findings is that they are based on a single representative strain, Va8, selected for its moderate–high virulence (DSL = 4.625) among the 33 isolates. Given the substantial variation in virulence observed within the Inner Mongolia *V. alfalfae* population—with DSL values ranging from 3.04 (Va30) to 4.79 (Va23)—it is plausible that sensitivity to fungicides also varies among genetically and phenotypically diverse isolates and may arise from differences in baseline gene expression, target site polymorphisms, or efflux pump activity. Consequently, the EC_50_ values reported herein should be regarded as preliminary estimates that require validation through multi-isolate sensitivity assays encompassing strains with diverse virulence phenotypes and geographic origins. Nevertheless, these preliminary data provide an important baseline reference for prioritizing candidate fungicides and designing future population-level sensitivity assays. Such assays are essential to establish the full range of sensitivity within the regional *V. alfalfae* population and to determine whether the high efficacy of DMI-based fungicide combinations observed against Va8 is a general characteristic of this pathogen in Inner Mongolia.

It is essential to acknowledge the limitations inherent in translating in vitro fungicide sensitivity data to field-level disease control recommendations. The mycelial growth inhibition assay employed in this study evaluates only the direct toxicity of a fungicide to the pathogen under optimal growth conditions on artificial medium. It does not account for factors that critically influence field efficacy, including fungicide penetration into and translocation within the host plant xylem, the stability and persistence of the active ingredient in the soil and rhizosphere environment, the timing of application relative to the infection window, and the potential for fungicide degradation or leaching under field conditions. For vascular wilt pathogens, which inhabit the xylem and are therefore physically protected from superficially applied chemicals, systemic mobility and xylem compatibility are essential product characteristics. Future research should prioritize controlled-environment and field efficacy trials to validate the promising in vitro activity of DMI-based fungicide combinations observed in this preliminary study. These trials should evaluate different application methods—including seed treatment, soil drench, and foliar spray—at various timings relative to inoculation and under representative field conditions. Additionally, the baseline sensitivity data generated in this study will serve as a valuable reference against which potential shifts in fungicide sensitivity following the introduction of these products into routine use can be detected, forming an essential component of a proactive resistance monitoring strategy. Furthermore, multi-isolate sensitivity profiling should be conducted concurrently to establish robust baseline sensitivity data for the regional *V. alfalfae* population, which will serve as an essential reference for future resistance monitoring programs [34].

The substantial virulence continuum documented among the 33 *V. alfalfae* isolates (DSL 3.04–4.79) indicates that such phenotypically anchored collections are essential for moving beyond single-reference-genome comparisons toward pan-genomic analyses that can capture the lineage-specific effector repertoires, accessory genomic regions, and structural variants underlying quantitative virulence differences. In the context of the expanding alfalfa production systems in northern China, characterizing the microbial ecological niche of *V. alfalfae*—including potential synergistic, antagonistic, or neutral interkingdom interactions—will be critical for predicting disease risk trajectories and designing ecology-informed interventions.

In conclusion, this study establishes *V. alfalfae* as the causal agent of alfalfa Verticillium wilt in Inner Mongolia, representing a significant eastward range expansion of this pathogen within China. The disease is currently confined to Hohhot and Bayannur among the six regions surveyed, a finding that enables the targeted implementation of surveillance, quarantine, and containment measures before the pathogen becomes more widely established. The substantial variation in virulence among isolates provides a biological foundation for screening alfalfa germplasm for resistance against locally prevalent strains, while the in vitro fungicide sensitivity data from strain Va8 offer a preliminary reference point for prioritizing candidate fungicides for multi-isolate validation and subsequent field evaluation. Given the demonstrated capacity of *V. alfalfae* to spread through multiple pathways—irrigation water, soil movement, contaminated equipment, and infected planting material [35]—and the economic importance of alfalfa to the livestock industry of Inner Mongolia, sustained investment in pathogen monitoring, host resistance breeding, and integrated disease management research is both scientifically and economically justified. Collectively, the present study lays a foundational framework for establishing a robust integrated disease management (IDM) strategy against alfalfa Verticillium wilt. This comprehensive strategy encompasses targeted pathogen surveillance across high-risk regions, the rational deployment of resistant alfalfa cultivars adapted to locally dominant virulent isolates, and evidence-based fungicide application based on baseline sensitivity data of *V. alfalfae.* Future research priorities should focus on translating preliminary in vitro and greenhouse observations into practically feasible, field-validated management protocols, thereby mitigating the economic losses caused by this destructive disease across Inner Mongolia’s alfalfa production systems.

Future surveillance of *V. alfalfae* distribution and virulence dynamics in Inner Mongolia is imperative. Targeted monitoring in Hohhot, Bayannur, and adjacent seed-importing regions should be prioritized to enable early detection of range expansion. For resistance breeding, the predominance of moderate-to-high virulence isolates (DSL 3.9–4.6) necessitates screening alfalfa germplasm against regionally representative strains within this prevalent virulence class, rather than relying exclusively on reference strains from other provinces. The promising in vitro efficacy of DMI-based combinations requires validation through controlled-environment and field trials prior to management recommendations, with particular attention to application timings (e.g., seed treatment, soil drench) and xylem mobility under local edaphic conditions. Furthermore, nanocarrier–fungicide co-delivery represents an emerging strategy to enhance delivery efficiency and reduce environmental loading [35]. Although untested in alfalfa–Verticillium systems, this approach holds promise for improving xylem-targeted delivery of systemic fungicides against vascular wilt pathogens.

## Figures and Tables

**Table 1:** Geographic origins and isolate codes of *Verticillium alfalfae* strains collected from Hohhot and Bayannur, Inner Mongolia.

Location	Latitude (°N)	Longitude (°E)	Isolate Code
Loc1, Tumd Left Banner, Hohhot City	40.684258	111.303548	Va1 Va2 Va3 Va4 Va5 Va6 Va7 Va8 Va9 Va10
Loc2, Tumd Left Banner, Hohhot City	40.593588	111.776475	Va11 Va12 Va13 Va14 Va15 Va16 Va17 Va18 Va19 Va20 Va21 Va22 Va23 Va24 Va25 Va26
Loc3, Tumd Left Banner, Hohhot City	40.584181	111.781527	Va27 Va28
Loc4, Tumd Left Banner, Hohhot City	40.690442	111.333272	Va29 Va30
Loc5, Dengkou County, Bayannur League	40.515924	106.598325	Va31 Va32
Loc6, Tumd Left Banner, Hohhot City	40.55711	111.739240	J2-2

**Table 2:** Primers used for PCR amplification and sequencing in this study.

No.	Target Gene ^a^	Primer Name	Primer Sequencing (5′ to 3′)	T (°C)	Product Size (bp)	Reference
1	*act*	VActf	TAATTCACAATGGAGGGTAGG	48	588	[12]
		VActR	GTAAGGATACCACGCTTGG			
2	*gapdh*	VGPDf2	GGCATCAACGGTTTCGGCC	56	727	[12]
		VGPDr	GTAGGAGTGGACGGTGGTCATGAG			
3	*ts*	VTs3f	GCGCTGCAAGGCCGAGAAC	59	604	[12]
		VTs3r	GCGGAACGAGACGGCCTCC			
4	*tef1-α*	VEFf	AACGTCGTCGTCATCGGCCACG	60	683	[12]
		VEFr	CCACGCTCACGCTCGGCCTT			

^a^ *act*: Actin; *gapdh*: Glyceraldehyde-3-phosphate dehydrogenase; *ts*: Tryptophan synthase; *tef1-α*: Elongation factor 1-α.

**Table 3:** Information on the eight fungicides evaluated for in vitro activity against *Verticillium alfalfae* strain Va8.

Fungicide Name	Formulation ^a^	Registration Number	Plate Concentration (mg/L)
32.5% Difenoconazole	SC	PD20150707	4.096, 1.024, 0.256, 0.064, 0.016
10% Trifloxystrobin & 20% Tebuconazole	SC	PD20184323	0.256, 0.128, 0.064, 0.032, 0.016
30% Difenoconazole–Propiconazole	EC	PD20211385	0.256, 0.128, 0.064, 0.032, 0.016
15% Triadimefon	WP	PD20040283	16, 8, 4, 2, 1
32.5% Fluoxapiprolin Difenoconazole	SC	PD20220609	4.096, 1.024, 0.256, 0.064, 0.016
68% MetalaxyI-M Mancozeb	WG	PD20080846	320, 160, 80, 40, 20
70% Mancozeb	WP	PD20060179	128, 64, 32, 16, 8
35% Metalaxyl-M Fludioxonil	SD	PD20171139	128, 32, 8, 2, 0.5

^a^ SC, suspension concentrate; EC, emulsifiable concentrate; WP, wettable powder; WG, water-dispersible granule; SD, seed dressing.

**Table 4:** Pathogenicity of *Verticillium alfalfae* isolates on alfalfa seedlings (cv. Zhongmu No. 1) as assessed by disease severity level at 30 days post-inoculation.

Isolate	DSL (Mean ± SD)	Significant Difference (*p* = 0.05)	Significant Difference (*p* = 0.01)
Va23	4.792 ± 0.083	a	A
Va5	4.750 ± 0.096	ab	A
Va20	4.750 ± 0.289	ab	A
Va21	4.708 ± 0.160	abc	AB
Va32	4.667 ± 0.272	abc	ABC
Va8	4.625 ± 0.160	abc	ABCD
Va15	4.625 ± 0.160	abc	ABCD
Va24	4.625 ± 0.210	abc	ABCD
Va25	4.625 ± 0.285	abc	ABCD
Va19	4.583 ± 0.397	abcd	ABCDE
Va10	4.542 ± 0.210	abcde	ABCDEF
Va27	4.542 ± 0.344	abcde	ABCDEF
Va26	4.500 ± 0.333	abcdef	ABCDEF
Va1	4.458 ± 0.285	abcdefg	ABCDEF
Va13	4.458 ± 0.083	abcdefg	ABCDEF
Va22	4.458 ± 0.210	abcdefg	ABCDEF
Va2	4.417 ± 0.347	abcdefgh	ABCDEF
Va29	4.375 ± 0.250	abcdefgh	ABCDEF
Va7	4.333 ± 0.236	abcdefgh	ABCDEF
Ms198	4.333 ± 0.491	abcdefgh	ABCDEF
Va4	4.292 ± 0.394	abcdefgh	ABCDEFG
Va9	4.292 ± 0.083	abcdefgh	ABCDEFG
Va3	4.292 ± 0.479	abcdefgh	ABCDEFG
Va18	4.292 ± 0.438	abcdefgh	ABCDEFG
Va17	4.250 ± 0.215	bcdefgh	ABCDEFG
Va6	4.208 ± 0.160	cdefgh	ABCDEFG
Va14	4.208 ± 0.370	cdefgh	ABCDEFG
Va11	4.083 ± 0.441	defghi	BCDEFG
Va31	4.042 ± 0.083	efghi	CDEFG
Va28	4.000 ± 0.136	fghi	DEFG
Va12	3.958 ± 0.344	ghi	EFG
J2-2	3.917 ± 0.096	hi	FG
Va16	3.667 ± 0.527	-	G
Va30	3.042 ± 0.516	-	H

Note: a–i: Means followed by the same lowercase letter within a column are not significantly different at *p* = 0.05; A–H: Means followed by the same uppercase letter within a column are not significantly different at *p* = 0.01, according to Duncan’s multiple range test.

**Table 5:** In vitro toxicity of eight fungicides against *Verticillium alfalfae* strain Va8.

Fungicide Name	Treatment Concentration (ppm)	Concentration Log (x)	Probability Value (y)	Virulence Regression Equation	EC_50_ (μg/mL)	R^2^
32.5% Difenoconazole	4.096	0.61	5.9996	y = 1.063x + 5.22	0.62	0.9896
	1.024	0.01	5.1232			
	0.256	−0.59	4.4901			
	0.064	−1.19	3.9618			
	0.016	−1.80	3.3803			
10% Trifloxystrobin & 20% Tebuconazole	0.256	−0.59	5.2525	y = 1.9479x + 6.369	0.2	0.9975
	0.128	−0.89	4.5540			
	0.064	−1.19	4.0782			
	0.032	−1.49	3.4714			
	0.016	−1.80	2.8619			
32.5% Fluoxapiprolin Difenoconazole	4.096	0.61	5.9881	y = 1.1294x + 5.3681	0.47	0.9896
	1.024	0.01	5.3479			
	0.256	−0.59	4.8781			
	0.064	−1.19	4.0446			
	0.016	−1.80	3.2400			
30% Difenoconazole–Propiconazole	0.256	−0.59	5.2324	y = 1.9952x + 6.6849	0.14	0.9253
	0.128	−0.89	4.9972			
	0.064	−1.19	4.6854			
	0.032	−1.49	3.7446			
	0.016	−1.80	2.8556			
15% Triadimefon	16	1.20	5.2809	y = 1.7038x + 3.3062	9.86	0.9754
	8	0.90	4.8564			
	4	0.60	4.3787			
	2	0.30	3.9984			
	1	0.00	3.1454			
35% Metalaxyl-M Fludioxonil	128	2.11	5.0295	y = 0.6332x + 3.7253	103.04	0.9071
	32	1.51	4.5288			
	8	0.90	4.4716			
	2	0.30	4.1367			
	0.5	−0.30	3.3193			
70% Mancozeb	128	2.11	5.5820	y = 1.7444x + 1.9316	57.41	0.9916
	64	1.81	5.0622			
	32	1.51	4.5810			
	16	1.20	4.1468			
	8	0.90	3.4141			
68% Metalaxyl-M Mancozeb	320	2.51	5.0875	y = 1.3276x + 1.7278	288.40	0.9958
	160	2.20	4.6502			
	80	1.90	4.2160			
	40	1.60	3.8160			
	20	1.30	3.5063			

## Data Availability

The original contributions presented in this study are included in the article/Supplementary Material. Further inquiries can be directed to the corresponding author.

## Supplementary Materials

The following supporting information can be downloaded at: https://www.mdpi.com/article/10.3390/microorganisms14071394/s1, Figure S1: Geographic distribution of *Verticillium alfalfae* isolation locations in Inner Mongolia, China. Figure S2: Representative images of in vitro mycelial growth inhibition of Verticillium alfalfae strain Va8 on fungicide-amended PDA plates.
