# Supplementary material for: Occurrence and Characterization of Verticillium alfalfae Causing Alfalfa Verticillium Wilt in Inner Mongolia, China, with Preliminary Fungicide Sensitivity Assessment
Source: Microorganisms. 2026 Jun 24;14(7):1394. doi: 10.3390/microorganisms14071394 (PMC13413679; doi:10.3390/microorganisms14071394)
Supplement: Supplementary file 1 [file microorganisms-14-01394-s001.zip › Supplementary Figure S1.pdf]

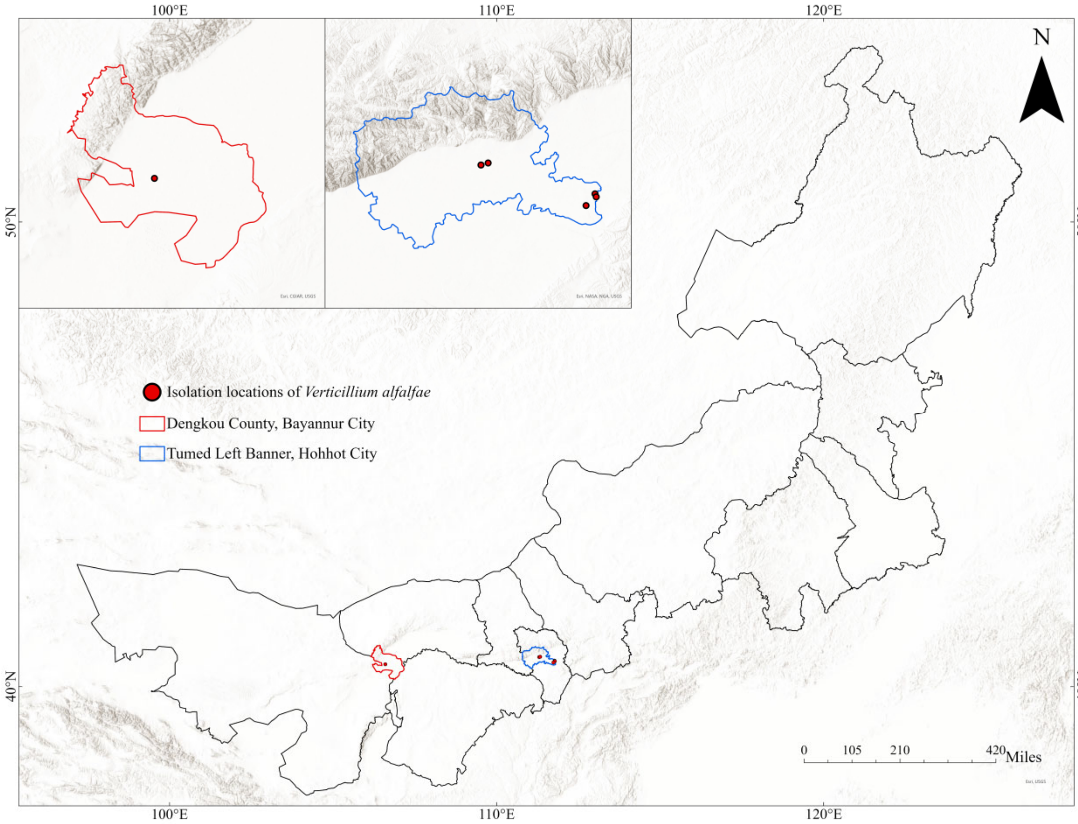

Supplementary Figure S1. Geographic distribution of *Verticillium alfalfae* isolation locations in Inner Mongolia, China. The main map shows the two administrative regions where the pathogen was successfully isolated: Dengkou County, Bayannur City (red boundary) and Tumd Left Banner, Hohhot City (blue boundary). Enlarged inset maps display the six specific sampling sites (red dots) within each region. The coordinates of all isolation locations are provided in Table 1.
