# Supplementary material for: Occurrence and Characterization of Verticillium alfalfae Causing Alfalfa Verticillium Wilt in Inner Mongolia, China, with Preliminary Fungicide Sensitivity Assessment
Source: Microorganisms. 2026 Jun 24;14(7):1394. doi: 10.3390/microorganisms14071394 (PMC13413679; doi:10.3390/microorganisms14071394)
Supplement: Supplementary file 1 [file microorganisms-14-01394-s001.zip › Supplementary Figure S2.pdf]

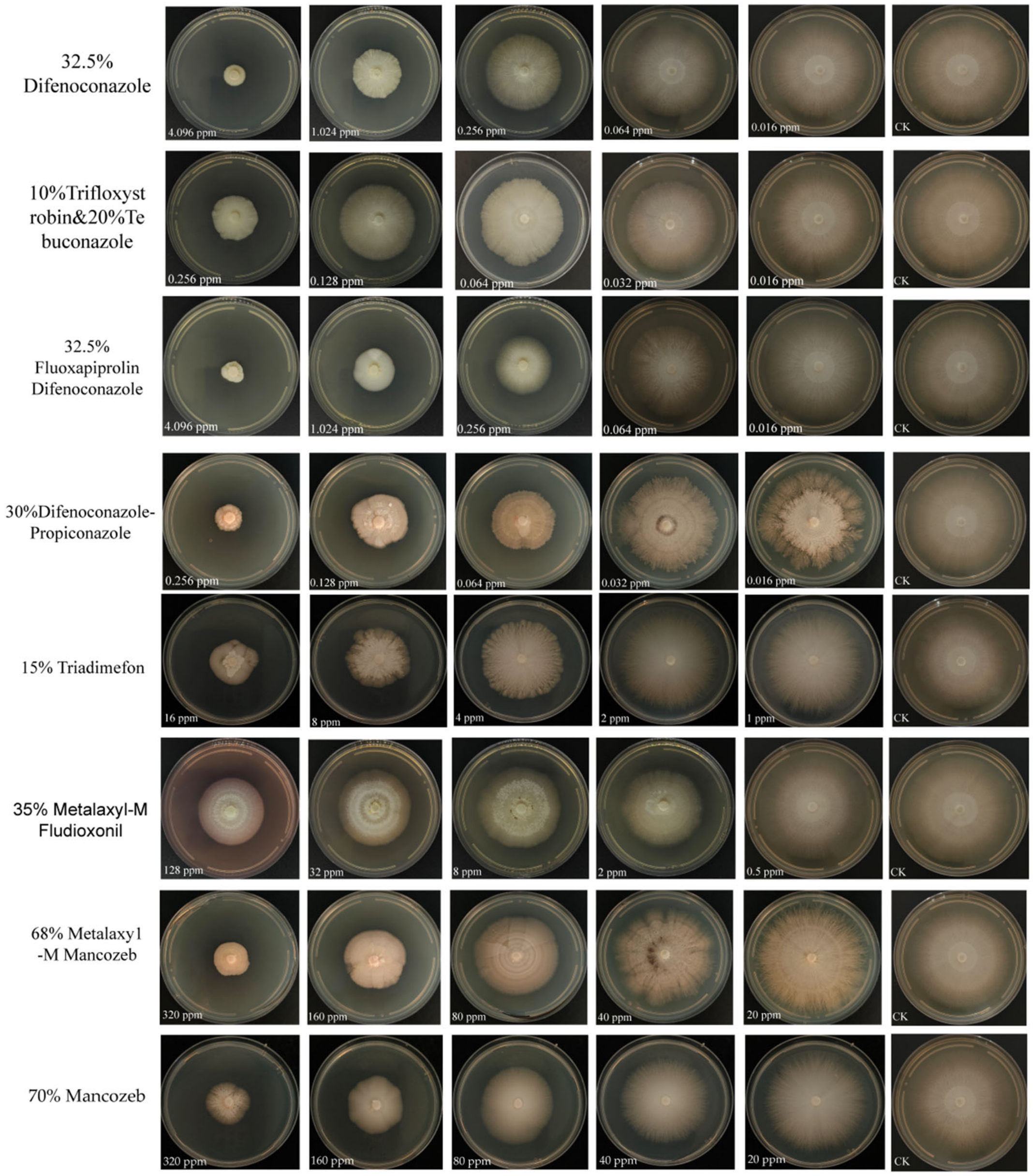

Supplementary Figure S2. Representative images of in vitro mycelial growth inhibition of Verticillium alfalfae strain Va8 on fungicide-amended PDA plates. Each row corresponds to one of the eight tested fungicides, with plates arranged from left to right at descending concentrations followed by an unamended negative control (CK). Top to bottom: 32.5% Difenoconazole (4.096, 1.024, 0.256, 0.064, 0.016 ppm); 10% Trifloxystrobin & 20% Tebuconazole (0.256, 0.128, 0.064, 0.032, 0.016 ppm); 32.5% Fluoxapiprolin & Difenoconazole (4.096, 1.024, 0.256, 0.064, 0.016 ppm); 30% Difenoconazole-Propiconazole (0.256, 0.128, 0.064, 0.032, 0.016 ppm); 15% Triadimefon (16, 8, 4, 2, 1 ppm); 35% Metalaxyl-M & Fludioxonil (128, 32, 8, 2, 0.5 ppm); 68% Metalaxyl-M & Mancozeb (320, 160, 80, 40, 20 ppm); and 70% Mancozeb (320, 160, 80, 40, 20 ppm). All plates were incubated at 25°C in darkness for 7–8 days.
